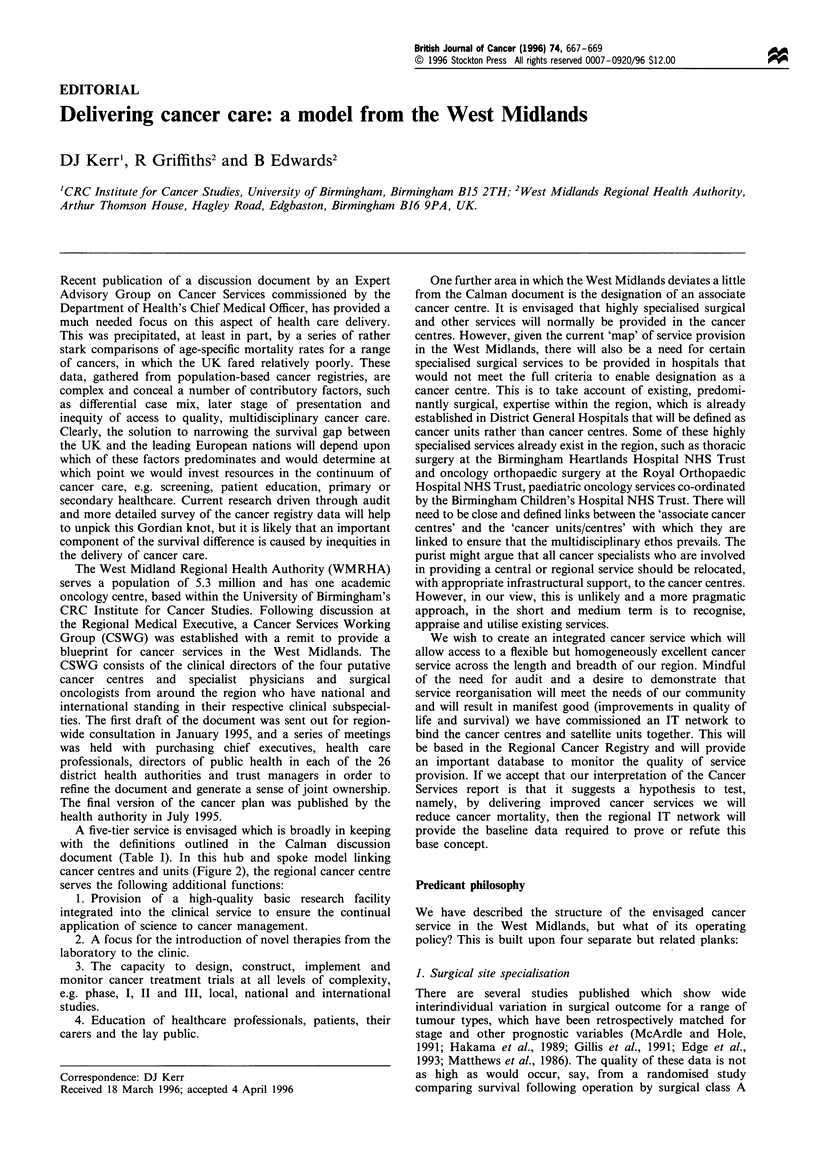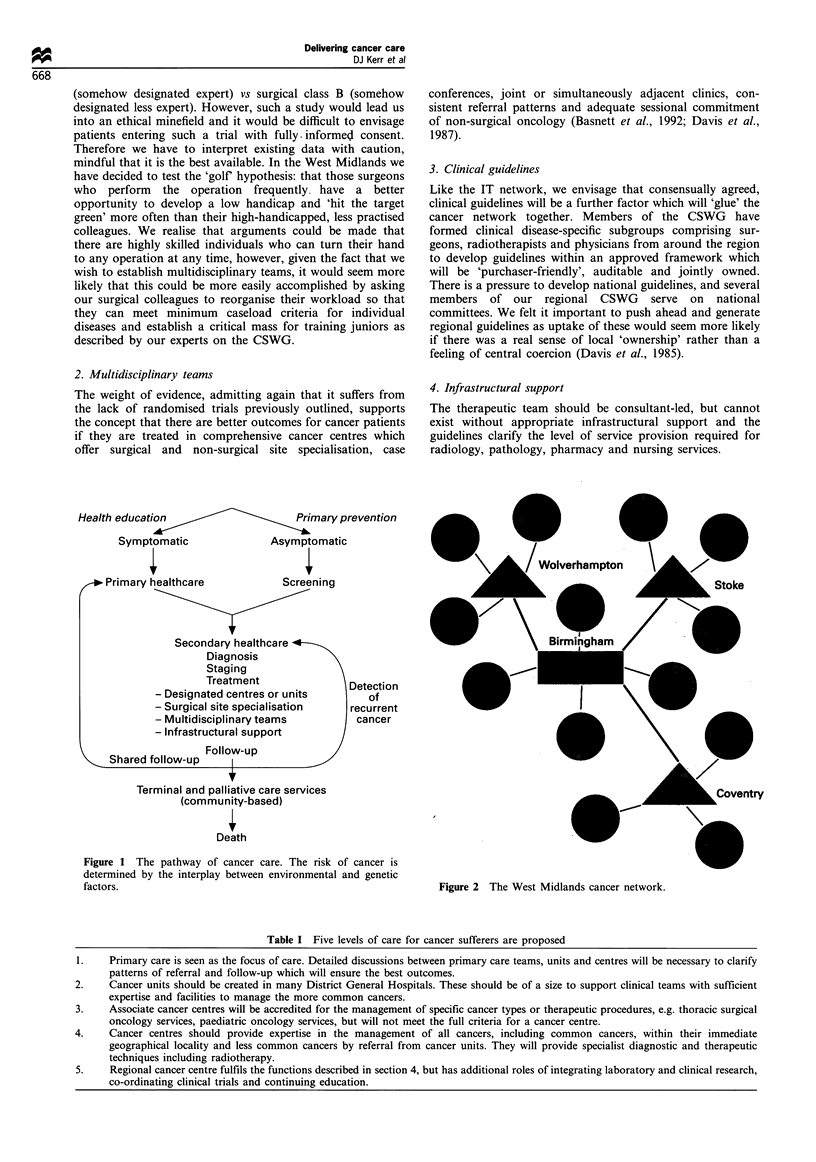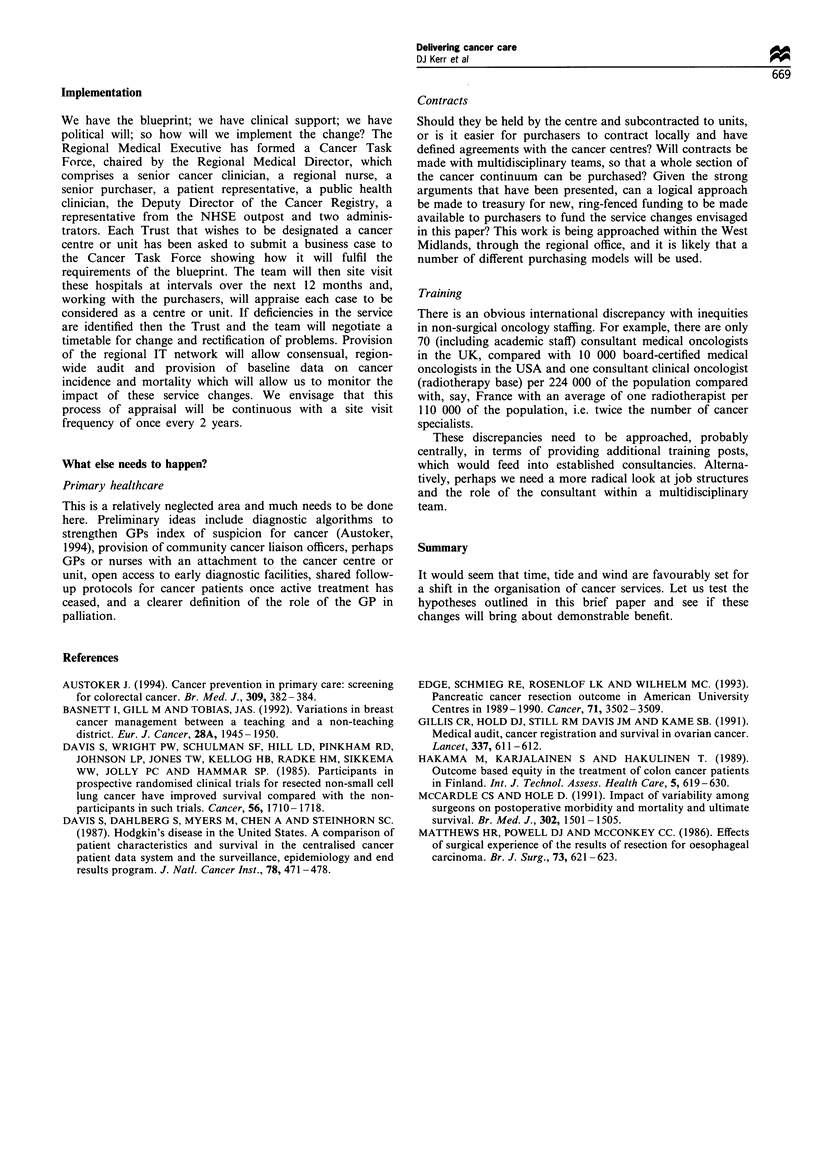# Delivering cancer care: a model from the West Midlands.

**DOI:** 10.1038/bjc.1996.419

**Published:** 1996-09

**Authors:** D. J. Kerr, R. Griffiths, B. Edwards

## Abstract

It would seem that time, tide and wind are favourably set for a shift in the organisation of cancer services. Let us test the hypotheses outlined in this brief paper and see if these changes will bring about demonstrable benefit.


British Journal of Cancer (1996) 74, 667-669
© 1996 Stockton Press All rights reserved 0007-0920/96 $12.00

EDITORIAL

### Delivering cancer care: a model from the West Midlands

DJ Kerr[1], R Griffiths[2] and B Edwards[2]

[1]*CRC Institute for Cancer Studies, University of Birmingham, Birmingham B15 2TH;* [2]*West Midlands Regional Health Authority, Arthur Thomson House, Hagley Road, Edgbaston, Birmingham B16 9PA, UK.*

Recent publication of a discussion document by an Expert Advisory Group on Cancer Services commissioned by the Department of Health's Chief Medical Officer, has provided a much needed focus on this aspect of health care delivery. This was precipitated, at least in part, by a series of rather stark comparisons of age-specific mortality rates for a range of cancers, in which the UK fared relatively poorly. These data, gathered from population-based cancer registries, are complex and conceal a number of contributory factors, such as differential case mix, later stage of presentation and inequity of access to quality, multidisciplinary cancer care. Clearly, the solution to narrowing the survival gap between the UK and the leading European nations will depend upon which of these factors predominates and would determine at which point we would invest resources in the continuum of cancer care, e.g. screening, patient education, primary or secondary healthcare. Current research driven through audit and more detailed survey of the cancer registry data will help to unpick this Gordian knot, but it is likely that an important component of the survival difference is caused by inequities in the delivery of cancer care.

The West Midland Regional Health Authority (WMRHA) serves a population of 5.3 million and has one academic oncology centre, based within the University of Birmingham's CRC Institute for Cancer Studies. Following discussion at the Regional Medical Executive, a Cancer Services Working Group (CSWG) was established with a remit to provide a blueprint for cancer services in the West Midlands. The CSWG consists of the clinical directors of the four putative cancer centres and specialist physicians and surgical oncologists from around the region who have national and international standing in their respective clinical subspecialties. The first draft of the document was sent out for region-wide consultation in January 1995, and a series of meetings was held with purchasing chief executives, health care professionals, directors of public health in each of the 26 district health authorities and trust managers in order to refine the document and generate a sense of joint ownership. The final version of the cancer plan was published by the health authority in July 1995.

A five-tier service is envisaged which is broadly in keeping with the definitions outlined in the Calman discussion document (Table I). In this hub and spoke model linking cancer centres and units (Figure 2), the regional cancer centre serves the following additional functions:

1. Provision of a high-quality basic research facility integrated into the clinical service to ensure the continual application of science to cancer management.
2. A focus for the introduction of novel therapies from the laboratory to the clinic.
3. The capacity to design, construct, implement and monitor cancer treatment trials at all levels of complexity, e.g. phase, I, II and III, local, national and international studies.
4. Education of healthcare professionals, patients, their carers and the lay public.

Correspondence: DJ Kerr
Received 18 March 1996; accepted 4 April 1996

One further area in which the West Midlands deviates a little from the Calman document is the designation of an associate cancer centre. It is envisaged that highly specialised surgical and other services will normally be provided in the cancer centres. However, given the current 'map' of service provision in the West Midlands, there will also be a need for certain specialised surgical services to be provided in hospitals that would not meet the full criteria to enable designation as a cancer centre. This is to take account of existing, predominantly surgical, expertise within the region, which is already established in District General Hospitals that will be defined as cancer units rather than cancer centres. Some of these highly specialised services already exist in the region, such as thoracic surgery at the Birmingham Heartlands Hospital NHS Trust and oncology orthopaedic surgery at the Royal Orthopaedic Hospital NHS Trust, paediatric oncology services co-ordinated by the Birmingham Children's Hospital NHS Trust. There will need to be close and defined links between the 'associate cancer centres' and the 'cancer units/centres' with which they are linked to ensure that the multidisciplinary ethos prevails. The purist might argue that all cancer specialists who are involved in providing a central or regional service should be relocated, with appropriate infrastructural support, to the cancer centres. However, in our view, this is unlikely and a more pragmatic approach, in the short and medium term is to recognise, appraise and utilise existing services.

We wish to create an integrated cancer service which will allow access to a flexible but homogeneously excellent cancer service across the length and breadth of our region. Mindful of the need for audit and a desire to demonstrate that service reorganisation will meet the needs of our community and will result in manifest good (improvements in quality of life and survival) we have commissioned an IT network to bind the cancer centres and satellite units together. This will be based in the Regional Cancer Registry and will provide an important database to monitor the quality of service provision. If we accept that our interpretation of the Cancer Services report is that it suggests a hypothesis to test, namely, by delivering improved cancer services we will reduce cancer mortality, then the regional IT network will provide the baseline data required to prove or refute this base concept.

### Predicant philosophy

We have described the structure of the envisaged cancer service in the West Midlands, but what of its operating policy? This is built upon four separate but related planks:

### *1. Surgical site specialisation*

There are several studies published which show wide interindividual variation in surgical outcome for a range of tumour types, which have been retrospectively matched for stage and other prognostic variables (McArdle and Hole, 1991; Hakama *et al.*, 1989; Gillis *et al.*, 1991; Edge *et al.*, 1993; Matthews *et al.*, 1986). The quality of these data is not as high as would occur, say, from a randomised study comparing survival following operation by surgical class A

(somehow designated expert) *vs* surgical class B (somehow designated less expert). However, such a study would lead us into an ethical minefield and it would be difficult to envisage patients entering such a trial with fully informed consent. Therefore we have to interpret existing data with caution, mindful that it is the best available. In the West Midlands we have decided to test the 'golf' hypothesis: that those surgeons who perform the operation frequently have a better opportunity to develop a low handicap and 'hit the target green' more often than their high-handicapped, less practised colleagues. We realise that arguments could be made that there are highly skilled individuals who can turn their hand to any operation at any time, however, given the fact that we wish to establish multidisciplinary teams, it would seem more likely that this could be more easily accomplished by asking our surgical colleagues to reorganise their workload so that they can meet minimum caseload criteria for individual diseases and establish a critical mass for training juniors as described by our experts on the CSWG.

*2. Multidisciplinary teams*

The weight of evidence, admitting again that it suffers from the lack of randomised trials previously outlined, supports the concept that there are better outcomes for cancer patients if they are treated in comprehensive cancer centres which offer surgical and non-surgical site specialisation, case conferences, joint or simultaneously adjacent clinics, consistent referral patterns and adequate sessional commitment of non-surgical oncology (Basnett *et al.*, 1992; Davis *et al.*, 1987).

*3. Clinical guidelines*

Like the IT network, we envisage that consensually agreed, clinical guidelines will be a further factor which will 'glue' the cancer network together. Members of the CSWG have formed clinical disease-specific subgroups comprising surgeons, radiotherapists and physicians from around the region to develop guidelines within an approved framework which will be 'purchaser-friendly', auditable and jointly owned. There is a pressure to develop national guidelines, and several members of our regional CSWG serve on national committees. We felt it important to push ahead and generate regional guidelines as uptake of these would seem more likely if there was a real sense of local 'ownership' rather than a feeling of central coercion (Davis *et al.*, 1985).

*4. Infrastructural support*

The therapeutic team should be consultant-led, but cannot exist without appropriate infrastructural support and the guidelines clarify the level of service provision required for radiology, pathology, pharmacy and nursing services.

**Figure 1** The pathway of cancer care. The risk of cancer is determined by the interplay between environmental and genetic factors.

**Figure 2** The West Midlands cancer network.

**Table I** Five levels of care for cancer sufferers are proposed

| | |
|---|---|
| 1. | Primary care is seen as the focus of care. Detailed discussions between primary care teams, units and centres will be necessary to clarify patterns of referral and follow-up which will ensure the best outcomes. |
| 2. | Cancer units should be created in many District General Hospitals. These should be of a size to support clinical teams with sufficient expertise and facilities to manage the more common cancers. |
| 3. | Associate cancer centres will be accredited for the management of specific cancer types or therapeutic procedures, e.g. thoracic surgical oncology services, paediatric oncology services, but will not meet the full criteria for a cancer centre. |
| 4. | Cancer centres should provide expertise in the management of all cancers, including common cancers, within their immediate geographical locality and less common cancers by referral from cancer units. They will provide specialist diagnostic and therapeutic techniques including radiotherapy. |
| 5. | Regional cancer centre fulfils the functions described in section 4, but has additional roles of integrating laboratory and clinical research, co-ordinating clinical trials and continuing education. |

### Implementation

We have the blueprint; we have clinical support; we have political will; so how will we implement the change? The Regional Medical Executive has formed a Cancer Task Force, chaired by the Regional Medical Director, which comprises a senior cancer clinician, a regional nurse, a senior purchaser, a patient representative, a public health clinician, the Deputy Director of the Cancer Registry, a representative from the NHSE outpost and two administrators. Each Trust that wishes to be designated a cancer centre or unit has been asked to submit a business case to the Cancer Task Force showing how it will fulfil the requirements of the blueprint. The team will then site visit these hospitals at intervals over the next 12 months and, working with the purchasers, will appraise each case to be considered as a centre or unit. If deficiencies in the service are identified then the Trust and the team will negotiate a timetable for change and rectification of problems. Provision of the regional IT network will allow consensual, region-wide audit and provision of baseline data on cancer incidence and mortality which will allow us to monitor the impact of these service changes. We envisage that this process of appraisal will be continuous with a site visit frequency of once every 2 years.

### What else needs to happen?

### *Primary healthcare*

This is a relatively neglected area and much needs to be done here. Preliminary ideas include diagnostic algorithms to strengthen GPs index of suspicion for cancer (Austoker, 1994), provision of community cancer liaison officers, perhaps GPs or nurses with an attachment to the cancer centre or unit, open access to early diagnostic facilities, shared follow-up protocols for cancer patients once active treatment has ceased, and a clearer definition of the role of the GP in palliation.

### *Contracts*

Should they be held by the centre and subcontracted to units, or is it easier for purchasers to contract locally and have defined agreements with the cancer centres? Will contracts be made with multidisciplinary teams, so that a whole section of the cancer continuum can be purchased? Given the strong arguments that have been presented, can a logical approach be made to treasury for new, ring-fenced funding to be made available to purchasers to fund the service changes envisaged in this paper? This work is being approached within the West Midlands, through the regional office, and it is likely that a number of different purchasing models will be used.

### *Training*

There is an obvious international discrepancy with inequities in non-surgical oncology staffing. For example, there are only 70 (including academic staff) consultant medical oncologists in the UK, compared with 10 000 board-certified medical oncologists in the USA and one consultant clinical oncologist (radiotherapy base) per 224 000 of the population compared with, say, France with an average of one radiotherapist per 110 000 of the population, i.e. twice the number of cancer specialists.

These discrepancies need to be approached, probably centrally, in terms of providing additional training posts, which would feed into established consultancies. Alternatively, perhaps we need a more radical look at job structures and the role of the consultant within a multidisciplinary team.

### Summary

It would seem that time, tide and wind are favourably set for a shift in the organisation of cancer services. Let us test the hypotheses outlined in this brief paper and see if these changes will bring about demonstrable benefit.

### References

AUSTOKER J. (1994). Cancer prevention in primary care: screening for colorectal cancer. *Br. Med. J.*, **309**, 382–384.

BASNETT I, GILL M AND TOBIAS, JAS. (1992). Variations in breast cancer management between a teaching and a non-teaching district. *Eur. J. Cancer*, **28A**, 1945–1950.

DAVIS S, WRIGHT PW, SCHULMAN SF, HILL LD, PINKHAM RD, JOHNSON LP, JONES TW, KELLOG HB, RADKE HM, SIKKEMA WW, JOLLY PC AND HAMMAR SP. (1985). Participants in prospective randomised clinical trials for resected non-small cell lung cancer have improved survival compared with the non-participants in such trials. *Cancer*, **56**, 1710–1718.

DAVIS S, DAHLBERG S, MYERS M, CHEN A AND STEINHORN SC. (1987). Hodgkin's disease in the United States. A comparison of patient characteristics and survival in the centralised cancer patient data system and the surveillance, epidemiology and end results program. *J. Natl. Cancer Inst.*, **78**, 471–478.

EDGE, SCHMIEG RE, ROSENLOF LK AND WILHELM MC. (1993). Pancreatic cancer resection outcome in American University Centres in 1989–1990. *Cancer*, **71**, 3502–3509.

GILLIS CR, HOLD DJ, STILL RM DAVIS JM AND KAME SB. (1991). Medical audit, cancer registration and survival in ovarian cancer. *Lancet*, **337**, 611–612.

HAKAMA M, KARJALAINEN S AND HAKULINEN T. (1989). Outcome based equity in the treatment of colon cancer patients in Finland. *Int. J. Technol. Assess. Health Care*, **5**, 619–630.

MCCARDLE CS AND HOLE D. (1991). Impact of variability among surgeons on postoperative morbidity and mortality and ultimate survival. *Br. Med. J.*, **302**, 1501–1505.

MATTHEWS HR, POWELL DJ AND MCCONKEY CC. (1986). Effects of surgical experience of the results of resection for oesophageal carcinoma. *Br. J. Surg.*, **73**, 621–623.